# Landauer Resistivity Dipole at One-Dimensional Defect Revealed via near-Field Photocurrent Nanoscopy

**DOI:** 10.1021/acs.nanolett.5c00437

**Published:** 2025-04-10

**Authors:** Francesca Falorsi, Marco Dembecki, Christian Eckel, Monica Kolek Martinez de Azagra, Kenji Watanabe, Takashi Taniguchi, Martin Statz, R. Thomas Weitz

**Affiliations:** † First Institute of Physics, Faculty of Physics, 9375 Georg-August-University Göttingen, Göttingen 37077, Germany; ‡ Walter Schottky Institute, Technische Universität München, Garching 85748, Germany; § Research Center for Electronic and Optical Materials, 52747 National Institute for Materials Science, 1-1 Namiki, Tsukuba 305-0044, Japan; ∥ Research Center for Materials Nanoarchitectonics, 52747 National Institute for Materials Science, 1-1 Namiki, Tsukuba 305-0044, Japan; ⊥ International Center for Advanced Study of Energy Conversion, Göttingen ICASEC, Göttingen 37077, Germany

**Keywords:** Nanoscopic resistivity, Landauer resistivity dipole, Photocurrent imaging, s-SNOM, Graphene heterostructures

## Abstract

The fundamental question of how to describe ohmic resistance at the nanoscale was answered by Landauer in his seminal picture of the Landauer resistivity dipole (LRD). While this picture is theoretically well understood, experimental studies remain scarce due to the need for noninvasive local probes. Here, we use the nanometer lateral resolution of near-field photocurrent imaging to thoroughly characterize a monolayer–bilayer graphene interface. Via systematic tuning of charge carrier density and current flow, we detected charge carrier accumulation around this nearly ideal one-dimensional defect due to the formation of the LRDs. We found that, at low doping levels, the photocurrent exhibits the same polarity as the applied source–drain voltage, reflecting carrier concentration changes induced by the LRDs. This signature disappears at higher charge carrier densities in agreement with the numerical calculations performed. Photocurrent nanoscopy can thus serve as a noninvasive technique to study local dissipation at hidden interfaces.

## Graphical abstract

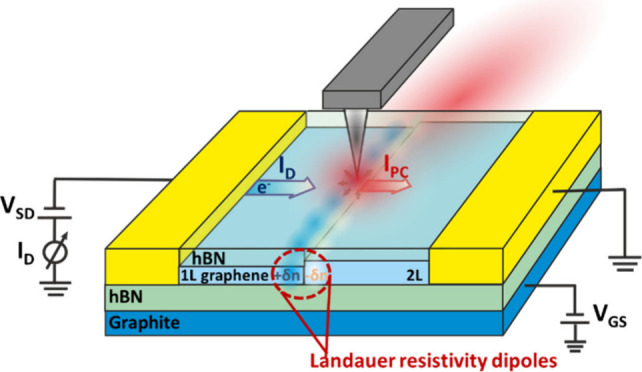

As the scaling of electrical integrated circuits down to a few nanometer sizes continues, the associated increase in current densities intensifies detrimental effects such as electromigration and excess heat generation.^1^ Consequently, understanding the precise mechanisms that govern electronic flow and resistivity on a nanoscopic level is a topic that continues to attract experimental and theoretical research.^2–5^ One fundamental mechanism of resistance at the nanoscale, proposed by Landauer, involves the creation of residual resistivity dipoles, often referred to as Landauer resistivity dipoles (LRDs).^6–9^ According to the LRD theory, localized dipoles form around defects during current flow, serving as a nanoscopic cause of resistance. Consequently, the voltage drop throughout the material is not homogeneous but rather highly concentrated around these defects.

To add further understanding to this picture, it would be helpful to directly observe these dipoles. The spatial long-range dipolar electric field generated by the LRD is challenging to access experimentally in three dimensions, due to its inherently low dipole strength.^10^ It was recognized early on^9^ that, in two dimensions, the spatial extent of the LRD is larger than in 3D, due to decreased screening.^10^ In general, the local potential build-up by the LRD in 2D is given by

$$V(r)\approx p\;\left(\frac{\cos(r)}{r}\right)$$

where *p* is the dipole moment and *θ* is the angle between *r* (the distance from the scatterer) and the current.^9^ In the diffusive limit, the total electric field caused by the dipoles surrounding the scatterer, defined as *E* ^dip^ = *n*_s_ *p*, is equal to the Ohmic electric field across the sample. Consequently, the LRD dipole strength is given by

$$p\approx \frac{j}{\left(\sigma n_{s}\right)}$$

where *σ* is the conductivity of the sample, *j* the incident current density, and *n*_s_ the density of scatterers.^7^ The potential buildup due to the LRD increases with current density and higher sample resistivity. Especially interesting are, therefore, 2D systems, such as van der Waals materials,^11–14^ in which both *σ* and *j* can be tuned in situ, making them an ideal system to study the LRD. Graphene,^15–17^ in particular, serves as an excellent platform to investigate the effects of scatterers on a local scale. Scatterers not only influence the local buildup of LRD but also affect the local dissipation,^18^ which can be elegantly imaged by scanning SQUID.^19,20^ However, the LRDs do not contribute relevantly to Joule heating, thus different imaging techniques are required. One approach involves scanning tunneling potentiometry (STP) and conductive AFM, which have been employed to study the LRD formation in 2D materials.

Indeed, the localized voltage drop across scatterers in graphene and monolayer (ML)/bilayer (BL) graphene interface junctions was experimentally measured on devices grown on silicon carbide using STP at low temperatures (ranging from 6 K to 80 K)^2,21–24^ and conductive AFM.^25^ In these studies, the Fermi energy is maintained constant. Ji et al.^24^ and Willke et al.^23^ showed that the localized voltage drop has a sign and magnitude related to the current flow. Similar measurements were done on *p*–*n* junctions of graphene on h-BN^4^ and on graphene with localized charged defects.^26^ While these fundamental studies show part of the expected response of the LRD at cryogenic temperatures, signatures of LRD under technologically relevant conditions (room temperature) or as a function of current under control of the Fermi energy (and with it, the conductivity), to the best of our knowledge, have not been performed.

Here, we discuss systematic investigations of a graphene ML to a Bernal-stacked BL interface as a function of electrostatically tuned charge carrier density (which controls *σ*) and current density *j* to locally identify the LRD. The wave function mismatch at this interface reduces electron transmission, allowing us to consider it as a reflective wall.^23^ To study the local buildup of carriers at the ML/BL interface as a function of σ and *j*, we use photocurrent imaging, which was successfully used to study lateral charge accumulation in van der Waals materials such as graphene.^27^ Governed by the photothermoelectric effect in graphene, this method is particularly sensitive to the local Seebeck coefficient and thus chemical potential.^28–30^ To allow for a nanoscale resolution, we use scanning near-field optical microscopy (SNOM)^31–33^ which enables photocurrent mapping at a scale equivalent to the tip radius (∼20 nm).^34,35^ High-resolution SNOM photocurrent nanoscopy in graphene systems has been used for the detection of propagating collective carrier modes,^36,37^ domain walls,^38–40^ and electronic transport phases in twisted graphene samples at low temperatures.^41,42^

We exploit the sensitivity of the photocurrent measured with SNOM to the local chemical potential gradient and use it to study the formation of LRD at ML/BL graphene interfaces during current flow under ambient conditions. We complement our experimental results with numerical calculations, which confirm that the formation of LRDs at the interface leads to a change in polarity of the photocurrent response upon changing the applied bias when the device is near its charge neutrality point (CNP), i.e., at low doping levels. This signature disappears at higher doping concentrations, where the device response is dominated by carrier type, with minimal influence from the LRDs. With our measurements, we are thus able to consistently map the density and current dependence of the LRD in real space.

We first explain the general mechanism by which a photocurrent (*I*_PC_) in graphene can emerge, before introducing our measurements. When electrons in graphene are excited by incident radiation, the absorbed photon energy is rapidly transformed into the formation of a hot Fermi distribution by electron–electron scattering. Due to the low electron–phonon coupling, electron cooling is inefficient, allowing hot electrons to remain decoupled from the lattice for relatively long times (from picoseconds up to milliseconds).^29,30,43,44^ The hot electrons can then produce a net photocurrent at local regions characterized by inhomogeneous profiles of the thermopower (i.e., Seebeck coefficient). In our specific case of the ML/BL interface, when we consider *x* to be the direction perpendicular to the interface, *I*_PC_ can be expressed as^35^

$$I_{\mathrm{PC}}=-\frac{1}{L}\int_{0}^{L}\sigma \left(x\right)S\left(x\right)\;\frac{\partial T}{\partial x}\;dx$$ 1

where σ­(*x*) is the spatially dependent conductivity, *T*(*x*) is the elevated electron temperature profile induced by the SNOM tip, *S*(*x*) is the spatially dependent Seebeck coefficient, and *L* is the distance between the source and drain contacts. In our case, if we consider the only source for a spatially varying Seebeck coefficient to be the ML/BL interface, the Seebeck coefficient can be modeled as a step function, and the photocurrent signal depends on the Seebeck coefficient difference across the interface (Δ*S* = *S*_BL_ – *S*_ML_).^45^

In the semiclassical Boltzmann formalism under the relaxation time approximation, the Seebeck coefficient (*S*) can be written as^46^

$$S=-\frac{1}{eT}\frac{\int \left(\varepsilon -\mu \right)\frac{\partial f}{\partial \varepsilon }\sigma \left(\varepsilon \right)}{\int \frac{\partial f}{\partial \varepsilon }\sigma \left(\varepsilon \right)},\mathrm{with}\;\sigma \left(\varepsilon \right)=e^{2}v{\left(\varepsilon \right)}^{2}\mathrm{DoS}\left(\varepsilon \right)\frac{\tau \left(\varepsilon \right)}{2}$$ 2

where *e* is the electron charge, *T* the temperature, *f* the Fermi distribution, σ­(ε) is the energy-dependent conductivity, and μ the chemical potential. The electrical conductivity σ depends on the electron velocity (*v*), the density of states (DoS), and the scattering time (τ).^46,47^ The chemical potential (μ) is a direct function of the local carrier density *n*, since, near the CNP, it holds that μ^mono^ = $\pm \hbar v_{F}\sqrt{\pi n}$ ^16^ and μ^bi^ = $\pm \frac{\hbar^{2}n\pi }{2m}$ ,^48^ where *ℏ* is the reduced Planck’s constant, *v*_F_ is the Fermi velocity, and *m* is the effective mass of the electrons in bilayer graphene, which is defined as *m* ≅ 0.033 *m*_e_, with *m*_e_ being the electron mass.^49^ Consequently, the scanning photocurrent measurements, directly related to the local Seebeck coefficient, act as a sensitive probe for the local carrier density and, thus, the occurrence of the LRD.

The photocurrent measurements are performed using an SNOM with an illumination wavelength of λ = 10.551 μm. Integrated electrical measurement units enable photocurrent measurements parallel to the optical ones, and in-situ control of the source-drain (*V*_SD_) and back-gate (*V*_GS_) voltages applied to the system (Figure 1a). The locally detected photocurrent signal is demodulated with the first or second harmonic of the tip oscillation frequency (Ω), which allows the suppression of the far-field background signal.

**Figure 1:**
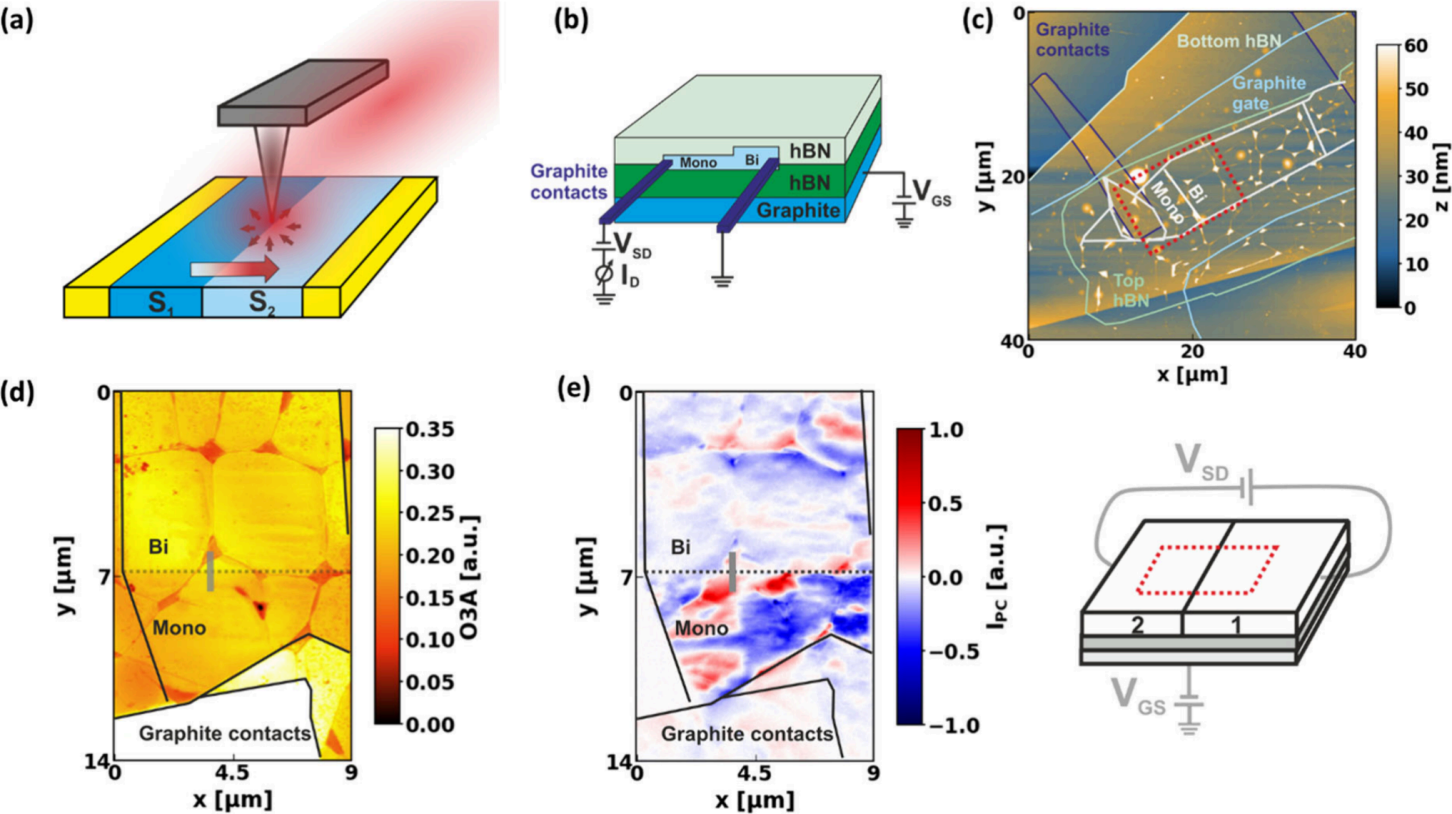
(a) Schematic representation of the setup used to analyze the spatial dependence of the photocurrent. (b) Schematic representation of the sample geometry. (c) AFM image of the sample studied with the different layers outlined. (d) 3^rd^ harmonic optical amplitude of area highlighted by the red rectangle in panel (c). (e) 2^nd^ harmonic photocurrent image, recorded simultaneously with the image in panel (d). The schematic on the right highlights the fact that, during the acquisition of the photocurrent map, neither *V*_GS_ nor *V*_SD_ are varied.

To analyze the carrier accumulation of impinging electrons at the ML/BL interface, the two main contacts for the source and drain are placed parallel to the interface. With this geometry, it is possible to probe the Seebeck coefficient gradient perpendicular to the current flow induced by the LRD.^40,50^ In both samples analyzed for this study, the graphene interface is encapsulated between two hexagonal boron nitride (hBN) flakes, the upper one being thinner than 7 nm, to retrieve the photocurrent signal originating from the underlying graphene.^34^ Graphite gates are used to improve signal quality and reduce energetic disorder in the active layers. The optical images of the used flakes are shown in Figure S2. A schematic of the investigated device structure is shown in Figure 1b, while AFM topography scans of the two samples considered for this study are shown in Figures 1c and Figure S3, where one can notice the cracks and bubbles created during the sample fabrication. Since the samples show similar results, the discussion below focuses on the sample shown in Figure 1c, while the complementary results of the second sample are shown in the Supporting Information.

Figures 1d and 1e show a spatial scan of the area highlighted by the dotted red rectangle in Figure 1c performed with the SNOM, Figure 1d shows the third harmonic optical amplitude image, while Figure 1e shows the second harmonic photocurrent signal that is recorded simultaneously. The schematic next to Figure 1e highlights the fact that, in the acquisition of this photocurrent signal, none of the other parameters that could be varied during the measurement, such as the *V*_GS_ or *V*_SD_, are adjusted; instead, the contacts are left floating. This scheme is maintained throughout the manuscript, with the main varying parameter of each respective photocurrent scan highlighted in red.

The photocurrent scan in Figure 1e shows that the main signal is generated by the spatial inhomogeneities of the sample, such as cracks or bubbles that are created during fabrication. In these regions, the carrier density and strain of the sample vary, resulting in a spatial texture of the Seebeck coefficient across the sample.^51,52^ In the rest of the work, we will focus on the photocurrent traces recorded perpendicular to the ML/BL interface, indicated by the faint gray line in Figure 1d. We use the interface as a well-localized one-dimensional (1D) defect for the investigation of the LRD. To establish the photocurrent measurements, we map (Figure 2a) the photocurrent as a function of charge carrier density, which is electrostatically controlled by the graphite gate (*V*_GS_). An additional small constant *V*_SD_ (1 mV) is applied to monitor the system resistance, which is shown for each point of the scan in the curve to the left of the scan. The dotted line indicates the position of the ML/BL interface. This position is obtained by aligning the photocurrent map with the third harmonic optical amplitude images, where the ML/BL interface is discernible by a difference in optical contrast, as shown, for example, in Figures S6 and S7. This alignment method is used throughout the manuscript.

**Figure 2:**
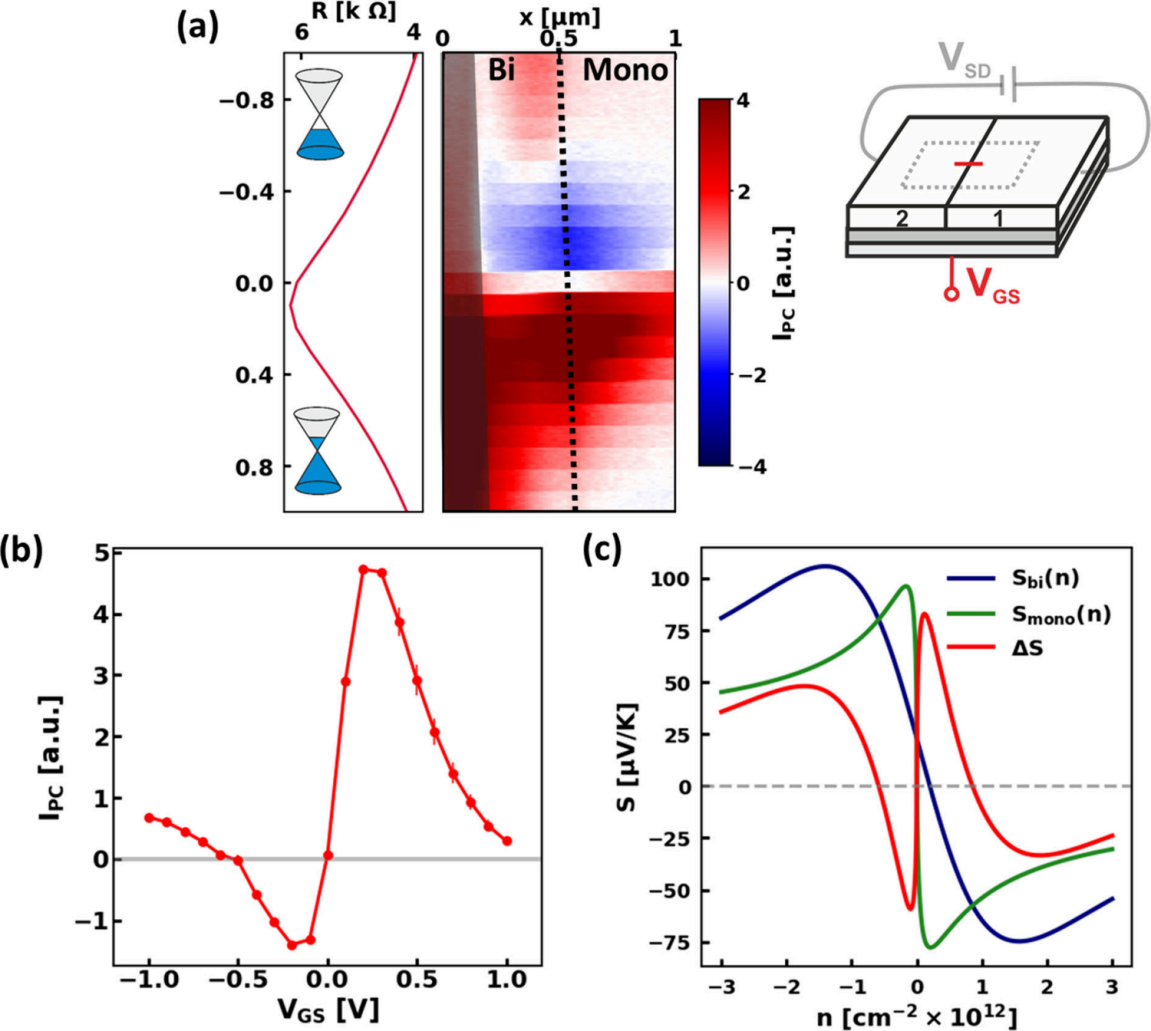
(a) 2^nd^ harmonic *I*_PC_ measured along a line across the ML/BL interface as a function of *V*_GS_. The value of *V*_GS_ varies from −1 V to 1 V, in steps of 0.1 V, with 10 line traces recorded for each step. The interface is marked by the black dotted line while under the gray shaded area on the left of the map a topographical defect is present. A potential of *V*_SD_ = 1 mV is applied to simultaneously monitor the resistance (graph to the left of the photocurrent scan). From the top to the bottom of this map, the transport changes from a hole to an electron-dominated transport, as schematically sketched with the Fermi level of the Dirac cones in the resistance plot. The schematic on the right highlights the fact that during the acquisition of this scan *V*_GS_ is varied while *V*_SD_ is maintained constant. (b) Vertical line cut of the photocurrent signal taken at the ML/BL interface. For each *V*_GS_, the average of the 10 *I*_PC_ values acquired at the interface for that *V*_GS_ is reported, with their standard deviation shown as an error bar. (c) Numerically calculated Seebeck coefficient of the ML, in green, and the BL in blue, with respect to the charge carrier density (at 300 K). The difference between the two Seebeck coefficients is shown in red.

As the gate voltage is tuned from negative to positive values, the induced carrier density transitions from holes to electrons, as shown in the schematic representation in Figure 2a. In this density region, the photocurrent near the interface exhibits a double sign switch, as highlighted by the line cut in Figure 2b, which is taken directly at the ML/BL interface. This behavior is related to the thermoelectric origin of the photocurrent, which depends on the difference in Seebeck coefficients between the ML and the BL graphene, Δ*S*.^45^ To understand the signal in more detail, we have numerically calculated the Seebeck coefficient of the ML, the BL, and the respective Δ*S* in Figure 2c. The Seebeck coefficient is simulated based on eq 2, without considering the density dependence of τ;^46,53,54^ more details are provided in S1. The simulated Δ*S* (Figure 2c) shows, as expected, the photocurrent’s density dependence with a double sign switch. The reason for this double sign switch is that the difference in band structure between ML and BL leads to a smoother transition of the Seebeck coefficient of the BL from electron- to hole-dominated transport, compared to the ML.^55,56^ The consistency of electrical measurements and simulations confirms the good understanding of the system.

The photocurrent signal has also a characteristic extent in the direction perpendicular to the interface. In Figure 2a, one can see that, after a maximum value around the CNP, the photocurrent in the ML decreases, and finally disappears for *V*_GS_ > 0.9 V on the electron and <-0.5 V on the hole side. In contrast, the photocurrent in the BL remains significant in the entire gate voltage window. The different spatial dependence of *I*_PC_ in the ML vs the BL arises because the *I*_PC_ is influenced not only by Δ*S* but also by the spatial extent of the temperature profile induced by the SNOM illumination (see eq 1). This profile is determined by the “cooling length”.^30,57^ The cooling length of the hot electrons (*L*_cool_) is defined as *L*_cool_ = $\sqrt{\frac{\kappa }{g}}$ , where *κ* is the thermal conductivity and *g* is a constant that accounts for the dispersion of the heat into the substrate.^35^ According to the Wiedemann–Franz law, κ is proportional to the conductivity of the sample.^58^ The lower resistance of the BL results in a longer *L*_cool_ than the ML (as shown in the measurements in Figure S5).

After having characterized the ML/BL in detail, we turn to the investigation of the LRD. The LRD potential at the interface translates into a local carrier buildup and, consequently, to a Δ*S* change, which we can locally measure as *I*_PC_. To investigate the local carrier accumulation at the interface due to the LRDs, the photocurrent response of the ML/BL interface is studied with respect to the applied bias. To this end, a line scan along the same position as in Figure 2a is performed with varying *I*_D_ at a fixed *V*_GS_. Figure 3a shows these scans for three constant *V*_GS_ values: in the hole-doped regime (*V*_GS_ = −100 mV), at the local CNP (*V*_GS_ = 0 V), and in the electron-doped regime (*V*_GS_ = 150 mV). We examine specifically the low-bias regime (|*V*_SD_| < 30 mV), where *I*_PC_ is generated by the PTE and is not expected to depend on *I*_D_. This is seen for scans taken in the doped regimes at *V*_GS_ = −100 mV and *V*_GS_ = 150 mV (Figure 3a). We note in passing that we can consequently also rule out that *I*_PC_ is due to the bolometric effect, where *I*_PC_ should indeed depend on the magnitude and sign of the applied bias^59–61^ (see Figure S7 for more details).

**Figure 3:**
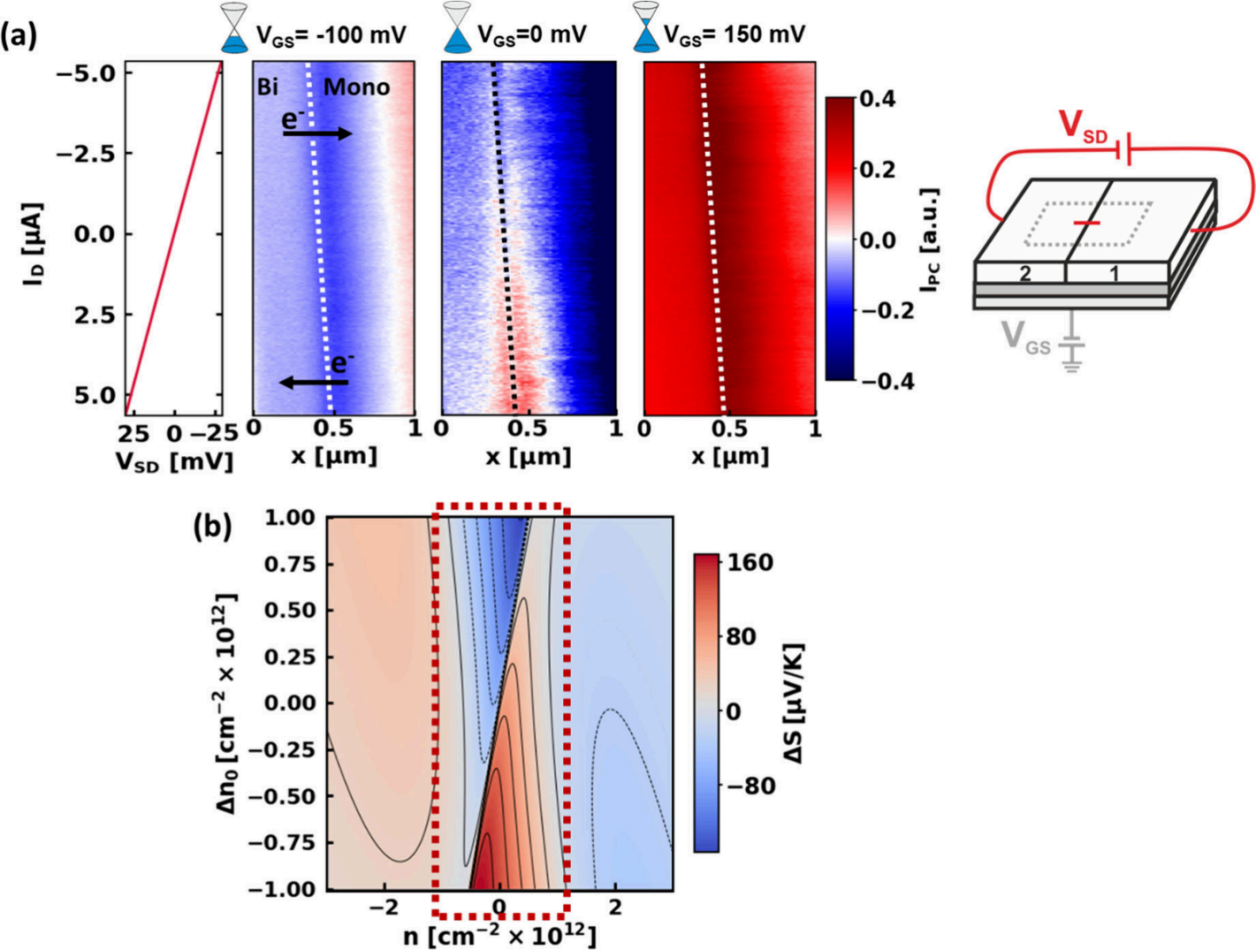
(a) 2^nd^ harmonic photocurrent maps measured along a line across the ML/BL interface (highlighted in Figure 1c), as a function of *I*_D_. The data are acquired with *V*_SD_ values ranging from −30 mV to 30 mV in steps of 2 mV, with 10 lines measured for each step. The three maps are measured for three different V_GS_ values, as indicated by the title on top of each map: *V*_GS_ = −100 mV, *V*_GS_ = 0 mV, and *V*_GS_ = 150 mV. The graph to the left of the maps shows the *I*_SD_, with respect to the *V*_SD_ value applied, recorded during the photocurrent map taken at *V*_GS_ = −100 mV; the *y*-axis and color bar are shared. The interface is indicated with the dotted lines. The arrows indicate the direction of electron flows respectively at the top and bottom halves of the maps, corresponding to the sign of the *V*_SD_. The schematic on the left highlights that the *V*_SD_ parameter is varied in the acquisition of these scans, while the *V*_GS_ parameter is maintained constant. (b) Simulated difference of the Seebeck coefficient value Δ*S* between the monolayer and the bilayer graphene, with respect to the total carrier density *n*, controlled by *V*_GS_ and an additional parameter Δ*n*_0_, independent of *n* and mimicking the role of the deviation of the carrier density *n* induced by the LRD around the interface, which is a consequence of the applied *I*_D_.

In contrast to these scans at high carrier density, a strong *I*_PC_ dependence on *I*_D_ is visible for the scan taken around the CNP (Figure 3a). We attribute this to the local formation of LRDs—due to the applied *I*_D_—which leads to a density difference Δ*n*_0_ across the interface and, with it, a Δ*S*, which we detect as localized *I*_PC_. This hypothesis is confirmed by numerical calculations (Figure 3b) of Δ*S* at the ML/BL junction, as a function of Δ*n*_0_ (caused by LRD *V*(*r*) ≈ *I*_D_) and *n* (controlled by *V*_BG_). In our calculations, we add, for each data point, Δ*n*_0_/2 carriers to the ML and −Δ*n*_0_/2 to the BL. Most interesting is the region within the dashed box (Figure 3b) near the CNP, where a sign change of *I*_D_ also leads to a sign change of Δ*S*, which should thus appear in *I*_PC_. This is expected, because near the CNP, a small Δ*n*_0_ is sufficient to induce electron transport on one side of the interface and hole transport on the other, leading to large differences in the Seebeck coefficients of the two regions (see Figure 2c). At higher *n*, the overall impact of the LRD induced Δ*n*_0_ on Δ*S* is comparatively small.

We have also analyzed the detailed *I*_PC_ (vs *I*_D_) dependence in a small *V*_GS_ window around the CNP in Figure 4. There, one can discern that *I*_PC_ is characterized by two main features: it is symmetric around the interface, with an intensity decreasing with the increasing distance from the interface, and it increases with higher *I*_D_. Furthermore, the *I*_D_ position for which the *I*_PC_ switches sign increases for *V*_GS_ further from the local CNP (around *V*_GS_ = −10 mV). This trend is highlighted by vertical line cuts at the positions indicated by the three arrows at the top of Figure 4a, shown in Figure 4b. These cuts compare the *I*_D_ dependence of the *I*_PC_ at a fixed *x*-position in the ML right at the interface at different *V*_GS_ and highlight that the *I*_D_ for which *I*_PC_ switches sign (i.e., the photocurrent values cross the *I*_PC_ = 0 line) depends on *V*_GS_. To better understand the spatial dependence of the *I*_PC_ vs *I*_D_, a simulation of spatial dependence of the photovoltage generated by a SNOM tip, with respect to different Δ*n*_0_ values, was performed (Figure 4c; see the Supporting Information for details). The simulated spatial scans are calculated for two different charge carrier densities of the system: *n* = 0 cm^–2^ and *n* = 0.4 × 10^12^ cm^–2^. The simulated scans in Figure 4c show the same spatial behavior as the scans in Figure 4a. Both for the simulations and measurements, the value for which the photocurrent switches sign shifts with the value of the total charge carrier density. While at *n* = 0 cm^–2^, the sign switch occurs precisely at Δ*n*_0_ = 0 cm^–2^; at *n* = 0.4 × 10^12^ cm^–2^, a strong negative shift of Δ*n*_0_ (to Δ*n*_0_ = −0.8 × 10^12^ cm^–2^) is necessary to observe the sign switch of the photocurrent. In the experiment, the Δ*n*_0_ accumulation at the BL/ML interface is a consequence of the LRD. Finally, the increase in *I*_PC_ with increasing Δ*n*_0_ is also consistent with the LRD picture where *V*(*r*) ≈ *I*.

**Figure 4:**
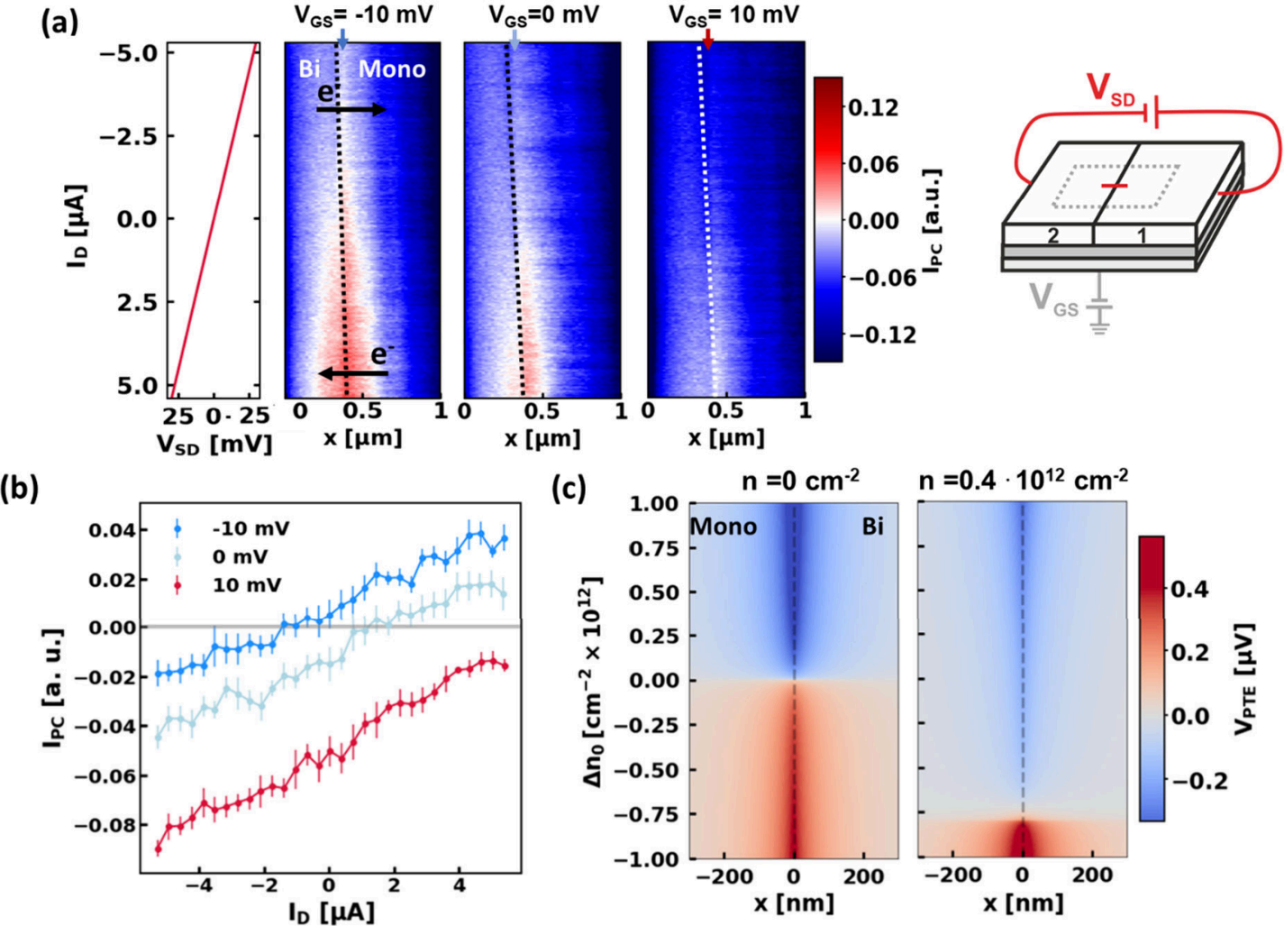
(a) 2^nd^ harmonic photocurrent maps measured across the interface, as a function of *I*_D_. The data are acquired with *V*_SD_ values ranging from −30 mV to 30 mV in steps of 2 mV, with 10 lines measured for each step. The three maps are measured for two different *V*_GS_ values around the CNP, as indicated by the title on top of each map: *V*_GS_ = −10 mV, *V*_GS_ = 0 mV, and *V*_GS_ = 10 mV. The graph to the left of the maps shows *I*_SD_ with respect to the *V*_SD_ applied, recorded during the photocurrent map taken at *V*_GS_ = −10 mV; the *y*-axis and color bar are shared. The interface is indicated with the dotted line and the arrows respectively indicate the direction of electron flow at the top and bottom halves of the maps, corresponding to the sign of the applied *V*_SD_. The schematic on the right indicates that *V*_SD_ is varied during the acquisition of these maps while *V*_GS_ is kept constant. (b) Vertical line cuts taken at the position in the ML indicated by the arrows in panel (a). For each *I*_D_, the average of the 10 *I*_PC_ values acquired at the arrow position for that *I*_D_ is reported, with their standard deviation shown as an error bar. (c) Numerically calculated spatial dependence of the photo thermoelectric voltage generated at a ML/BL interface (located at *x* = 0) varying the values of the parameter of Δ*n*_0_ for two different carrier density values: *n* = 0 cm^–2^ and *n* = 0.4 × 10^12^ cm^–2^. The spatial coordinate *x* is perpendicular to the interface and the parameter Δ*n*_0_ mimics the deviation of the carrier density *n* induced by the *I*_SD_-induced LRD around the interface.

In conclusion, the nanoscopic photocurrent detection based on a SNOM under ambient conditions allows the observation of Landauer resistivity dipoles in the electronic current flow and the direct visualization of the carriers accumulating at a ML/BL graphene interface, which due to the local wave function mismatch acts like a localized 1D defect. The possibility to analyze local, buried defects with a noninvasive method could be of use for the analysis of integrated circuits where local resistances could be detected prior to device failure.

## Supplementary Material



## Notes

Numerical simulations; optical images of sample 1, overview of sample 2, electrical characterization of samples, line scans of *I*_PC_ vs *V*_GS_ of sample 2, line scans of *I*_PC_ vs *V*_GS_ close to CNP for sample 1, line scans of *I*_PC_ vs *V*_SD_ far from CNP of sample 1, line scans of *I*_PC_ vs *iV*_SD_ far from CNP of sample 2, line scans of *I*_PC_ vs *V*_SD_ close to CNP of sample 2, experimental methods (PDF)
