# Correction to “Landauer Resistivity Dipole at One-Dimensional Defect Revealed via near-Field Photocurrent Nanoscopy”

**DOI:** 10.1021/acs.nanolett.6c00630

**Published:** 2026-03-04

**Authors:** Francesca Falorsi, Marco Dembecki, Christian Eckel, Monica Kolek Martinez de Azagra, Kenji Watanabe, Takashi Taniguchi, Martin Statz, R. Thomas Weitz

In the original publication, part of the acknowledgments are erroneously missing. The correct acknowledgments are displayed below.

