# Supplementary material for: Landauer Resistivity Dipole at One-Dimensional Defect Revealed via near-Field Photocurrent Nanoscopy
Source: Nano Lett. 2025 Apr 10;25(21):8495–502. doi: 10.1021/acs.nanolett.5c00437 (PMC12123675; doi:10.1021/acs.nanolett.5c00437)
Supplement: Supplementary file 1 [file nl5c00437_si_001.pdf]

Supplementary information

for

Landauer resistivity dipole at one dimensional defect revealed via near-field photocurrent nanoscopy

Francesca Falorsi[1], Marco Dembecki[2], Christian Eckel[1], Monica Kolek Martinez de Azagra[1], Kenji Watanabe[3], Takashi Taniguchi[4], Martin Statz[1], R. Thomas Weitz[1,5,*]

[1] 1st Institute of Physics, Faculty of Physics, Georg-August-University Göttingen, Göttingen 37077, Germany

[2] Walter Schottky Institute, Technische Universität München, Garching 85748, Germany

[3] Research Center for Electronic and Optical Materials, National Institute for Materials Science, 1-1 Namiki, Tsukuba 305-0044, Japan

[4] Research Center for Materials Nanoarchitectonics, National Institute for Materials Science, 1-1 Namiki, Tsukuba 305-0044, Japan

[5] International Center for Advanced Study of Energy Conversion, Göttingen ICASEC, Göttingen 37077, Germany

* corresponding author: thomas.weitz@uni-goettingen.de
* corresponding author: thomas.weitz@uni-goettingen.de

S1: Numerical simulation

A numerical simulation is performed to analyze the effect of the current flow on the photocurrent at the monolayer (ML)/ bilayer (BL) graphene interface junction. In order to avoid taking the resistivity value from real data, it was decided to calculate the photo voltage instead of the photocurrent. The photovoltage generated by a SNOM tip, depends on the photo thermoelectric effect, considering for simplicity a one dimensional system it is described by [1]:

$$V_{PTE} = -\frac{1}{L}\int_0^L S(x)\frac{\partial T}{\partial x}dx \tag{1}$$

Where *x* is the direction parallel to the direct line between the contacts, *T* is the electronic temperature and *S(x)* is the Seebeck coefficient of the device. In order to have a non-zero photocurrent signal, the Seebeck coefficient function *S* must be variable in space. When the Seebeck coefficient is a step function, as in the case studied in the manuscript where we analyzed the photovoltage between a ML and a BL of graphene, the photovoltage can be calculated with Equation 1 with ΔS for x >0 and - ΔS for x<0, with ΔS being the Seebeck coefficient difference between the two parts of the interface junction [1].

In the semiclassical Boltzmann transport formalism in the relaxation time approximation, the Seebeck coefficient can be expressed as:

$$S = -\frac{1}{eT}\frac{\int(\varepsilon-\mu)\frac{\partial f}{\partial \varepsilon}\sigma(\varepsilon)}{\int\frac{\partial f}{\partial \varepsilon}\sigma(\varepsilon)} \quad \text{with} \quad \sigma(\varepsilon) = e^2 v(\varepsilon)^2 DoS(\varepsilon)\frac{\tau(\varepsilon)}{2} \tag{2}$$

Where *e* is the electron charge, *T* is the electronic temperature, *f* is the Fermi distribution, $\sigma(\varepsilon)$ is the energy dependent conductivity and μ is the chemical potential. The electrical conductivity σ depends on the electron velocity *v,* the density of states *(DoS)*, and the scattering time τ [2].

The dependence of the Seebeck coefficient with respect to the charge carrier density *n* can be obtained by expressing μ as a function of *n*. Near the charge neutrality point the Fermi energy of the ML and BL graphene can be approximated as [3,4]:

$$E_F^{mono} = \pm\hbar v_F\sqrt{\pi n}$$

$$E_F^{bi} = \pm\frac{\hbar^2 n\pi}{2\,m} \tag{3}$$

Where $\hbar$ is the reduced Planck´s constant, v_F is the Fermi velocity and, m is the effective mass of the electrons in BL graphene, equal to $m \cong 0.033\, m_e$, with m_e being the electron mass [5]. Therefore, in this approximation the *DoS* of the ML can be written as $DoS_{mono}(\varepsilon) \sim |\varepsilon|$ while the electron velocity v_{mono} is independent of the energy ε. On the contrary, for the BL, the density of states can be approximated as

a constant of the energy, while the velocity $v(\varepsilon)_{bi} \sim \sqrt{(|\varepsilon|)}$. To complete the calculation of the Seebeck coefficient the energy dependent part of the scattering time needs to be considered, as the constant multiplication factor cancels out, appearing in both the numerator and denominator of the expression.

The scattering time is highly dependent on the dominant type of scattering in the system. In this case, two types of scattering were assumed for both ML and BL graphene: a constant scattering time τ_0 and a scattering time τ_1 inversely proportional to the energy of the system $\tau_1(\epsilon) = \tau_1/\epsilon$, which can represent the scattering induced by both neutral white noise short range disorder and acoustic phonons [6]. Using Matthiessen's rule one gets $\tau \approx (1 + \epsilon)^{-1}$. A more accurate calculation of the Seebeck coefficient should take into account the scattering of the electrons with the screened charge impurities in the substrate [2,7]. The approximation made for the scattering time together with the approximation of a constant *DoS* in the BL leads to a broadening of the obtained Seebeck coefficient with respect to those previously reported [8,9] and to an underestimation of the Seebeck coefficient of the BL. Since the relative intensity of the two Seebeck coefficients is important for the photocurrent, the Seebeck coefficient obtained for the BL is multiplied by a factor of 1.8 to make it comparable to that of the ML. This factor is chosen to ensure $S_{BL} > S_{ML}$ and so that the Seebeck coefficient difference $\Delta S = S_{BL} - S_{ML}$ can be comparable to the trend of the acquired photocurrent signal. The charge carrier dependence of the Seebeck coefficients for ML and BL graphene is shown in Figure 2c of the main manuscript. From these calculations, it was also possible to obtain the graph shown in Figure 3b, where the ΔS is calculated with respect to the total carrier density n and an additional parameter Δn_0. The parameter Δn_0 mimics the interfacial carrier accumulation induced by the formation of the LRD when current flows through the system and is thus controlled experimentally with V_{SD}.

The spatial dependence of the photocurrent with respect to the direction perpendicular to the interface, x, is also predicted. To complete the calculation of the photocurrent in (1), the thermal profile is calculated according to the formula [10]:

$$T(r) = T_0 K_0\left(\sqrt{\frac{r^2 + L_{spot}^2}{L_{cool}^2}}\right) \tag{4}$$

Where T_0 is the maximum rise in temperature, K_0 is the modified Bessel function of the second kind of zeroth-order, $r = \sqrt{x^2 + y^2}$ is the radius, L_{cool} is the cooling length [11], the characteristic length scale at which the temperature gradient decays and, L_{spot} accounts for the finite laser spot size, in our case equal to the size of a SNOM tip (~20 nm).

The cooling length is strongly dependent on the dominant scattering mechanism in the system. Since the dynamic electron screening depends on the carrier density of the system, the cooling length is also

expected to depend on the Fermi energy [2,12]. In this work we have assumed the same cooling length for ML and BL graphene, independent of the Fermi energy. Since the cooling length depends on the spatial extent of the signal, a cooling length of 400 nm is assumed in the system by evaluating the extent of the signal shown in Figure 3a. Considering a system with the interface located at x=0, with x being the direction perpendicular to the interface, the calculated spatial dependence of the temperature increase upon laser illumination is shown in Figure S1.1a.

Finally, the generated photo-voltage at a ML/BL interface after the excitation shown in Figure S1.1a and the is shown in Figure S1.1b. Both the temperature and the photo-voltage profile show a sharp peak at x=0, where the heat source is located, decaying asymptotically to zero.

Figure S1.2 shows the spatial dependence of the photovoltage generated by a SNOM at an interface layer graphene positioned at x=0, in dependence of different *n* values. This graph corresponds to the scan shown in Figure 2a. The calculated and the measured graph show the same trend. What is not captured in the simulation is the charge carrier dependency of the cooling length and the asymmetry of the cooling length of the ML and BL graphene.

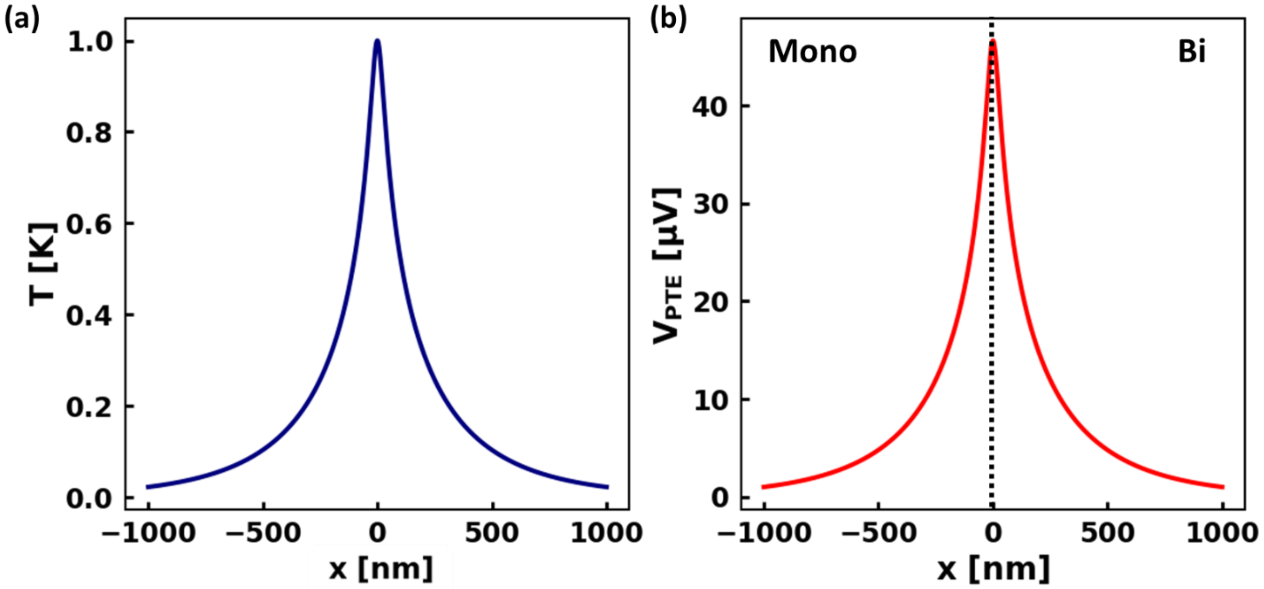

***Figure S1.1**: **(a)** Spatial dependence of the electronic temperature increase of a ML/BL graphene interface, with a SNOM tip located at x=0. Here x denotes the direction perpendicular to the interface. **(b)** Spatial dependence of the photothermoelectric voltage generated in the system described in (a).*

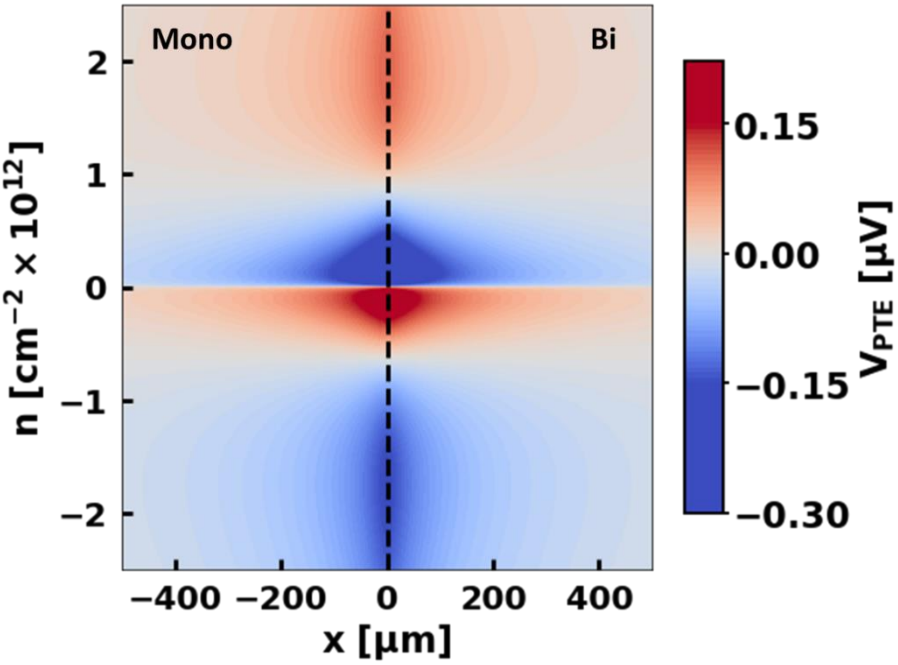

Figure S1.2*: Spatial dependence of the photo voltage increase of a ML/BL graphene interface in dependence of the total charge carrier density n.*

S2: Optical images of sample1

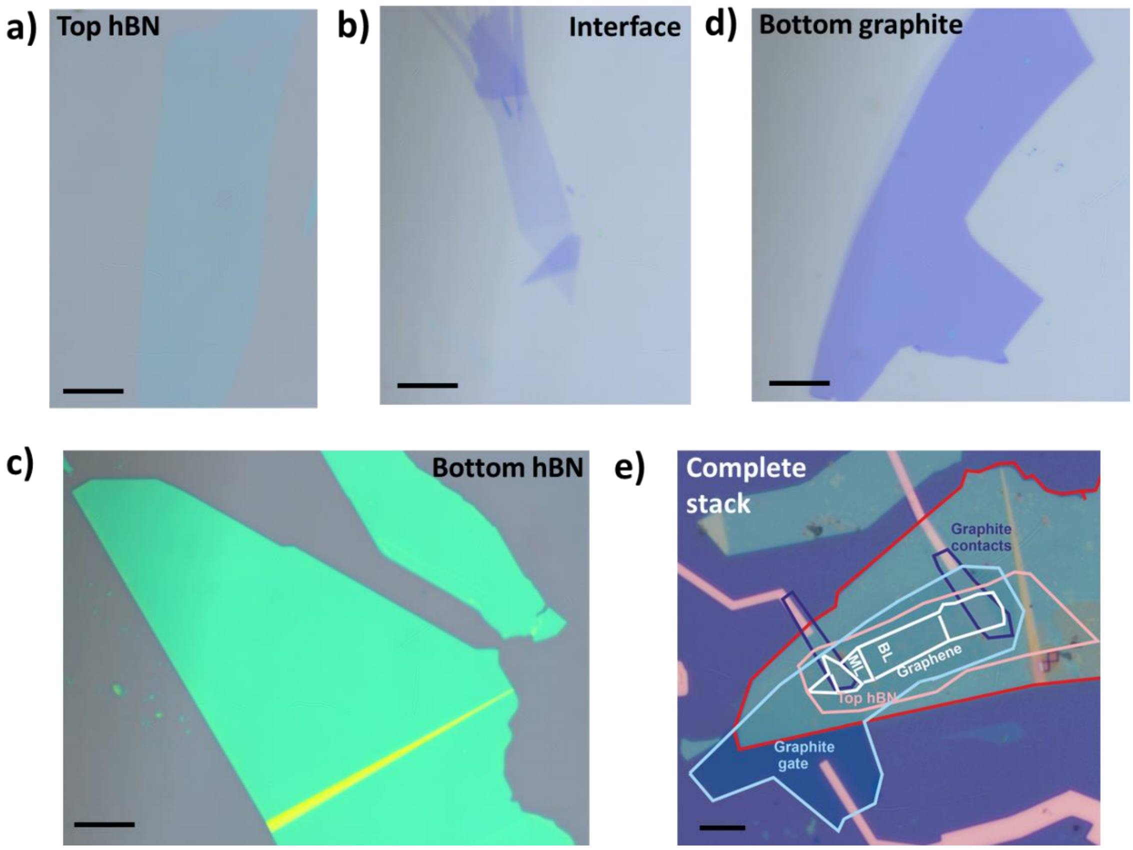

Figure S1: *Optical images of the main flakes used for the creation of the primary stack measured in the manuscript, scalebar=10 µm.* ***a)*** *Top hBN.* ***b)*** *ML/BL graphene interface junction.* ***c)*** *Bottom hBN.* ***d)*** *Bottom graphite.* ***e)*** *Optical image of the stack.*

S3: Sample 2

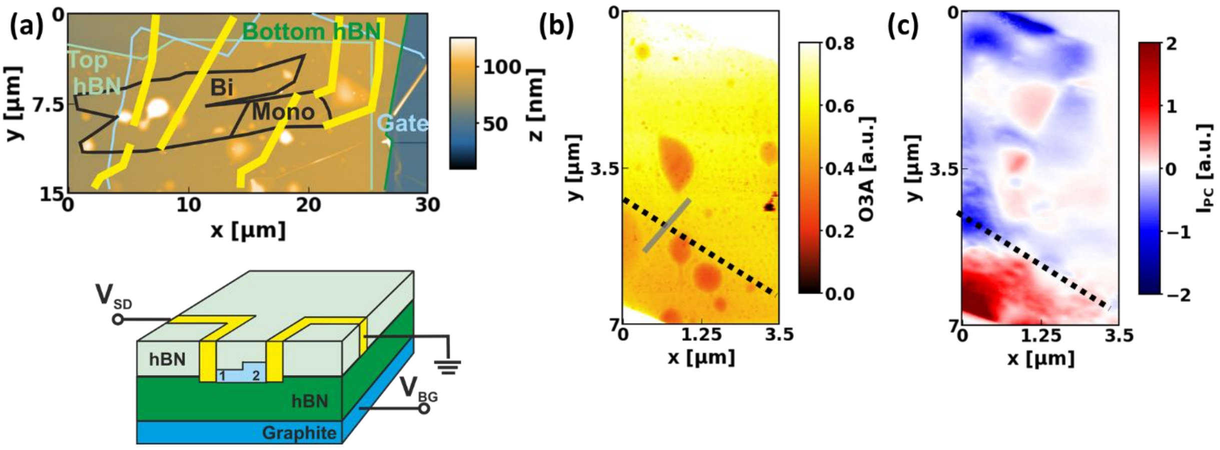

Figure S3: *Representation of the second sample analyzed.* ***(a)*** *Bottom: Schematic representation of the sample geometry. The ML/BL graphene interface is encapsulated in hBN, with a graphite gate used to tune the system's charge carrier density. The entire structure is situated on a 300 nm SiO_2/Si wafer. The electrical connection to the flakes is achieved through gold contacts etched through the top hBN layer. Top: An AFM image of the second sample, with different layers of the stack highlighted in various colors. The yellow lines represent contacts that were written onto the flake later. In this sample, in addition to the two primary contacts perpendicular to the interface (necessary to analyze the photocurrent dependence on the current impinging perpendicular to the interface), the ML and BL are also contacted separately.* ***(b)*** *3rd harmonic optical amplitude of the central part of the interface. Here, the interface is highlighted with the black dotted line and the gray line represents the line along which all line scans shown in Figures S8 and S9 were taken.* ***(c)*** *1st harmonic photocurrent map recorded simultaneously with (b). The photocurrent is generated mainly in inhomogeneous parts of the sample.*

S4: Electrical measurements

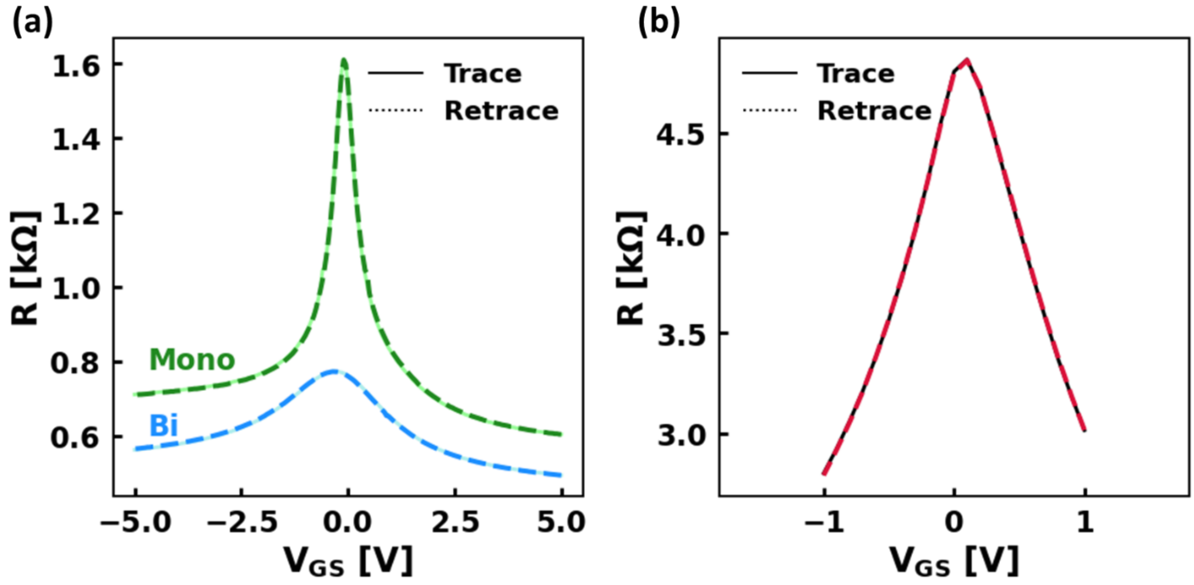

Figure S4: *Electrical characterization of the two samples studied.* ***(a)*** *Resistance between the two graphite contacts measured as a function of the back-gate voltage applied to sample 1, with a SD bias of 1 mV. The absence of hysteresis between the trace and retrace of the measurements indicates a lack of charge traps in the sample. Additionally, the sharpness of the peak and the low intrinsic doping (with the CNP located very close to* V_{GS}*=0) suggest a high degree of cleanliness and quality of the sample* [13]. ***(b)*** *Similar measurement for Sample 2, where the resistance is measured through the two additional gold contacts that separately contact the ML and BL graphene.*

S5: Gate sweep line scan of sample 2

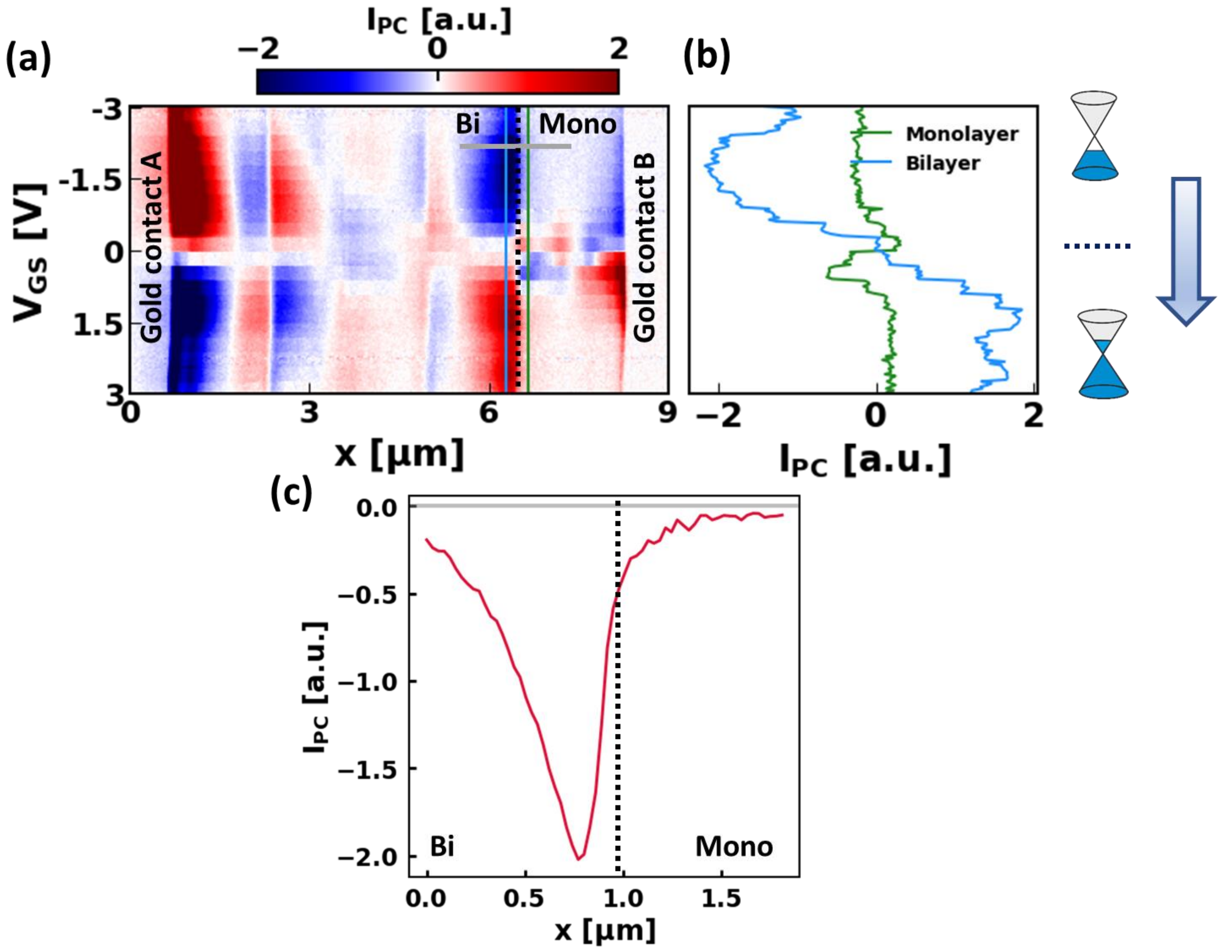

Figure S5: ***(a)*** *1st harmonic photocurrent along a line across contacts A and B (Figure S3.a) of sample 2 (x-axis) as a function of the applied back-gate voltage (y-axis). The* V_{GS} *varies from -3 V to 3 V in steps of 0.3 V, with 10 lines measured for each step. The interface is marked through the black dotted line. The 4-fold photocurrent behavior with respect to the applied gate-source voltage depends on the photothermoelectric effect being the main generation mechanism of the photocurrent. The other photocurrent signatures on the BL side, aside from the contacts and the interface, stem from local topographical inhomogeneities caused by the presence of bubbles and folds.* ***(b)*** *Vertical line cuts of the photocurrent values across the blue and green line in (a), with respect to a linear space between the maximum and the minimum* V_{GS} *of (a). The photocurrent in the ML rapidly decays to zero for gate voltages above 0.8 V, while the photocurrent in the BL remains significant up to 3 V. However, the photocurrent in the BL also begins to gradually diminish for* V_{GS} *values exceeding 1.5 V. These results are comparable to those obtained for Sample 1, as shown in Figure 2a.* ***(c)*** *Horizontal line cut across the gray line crossing the interface in (a). The spatial extension of the signal generated in the BL is longer than the one in the ML. This behavior is explained by the longer cooling length in the BL with respect to the ML.*

S6: Gate sweep line scan of Sample 1

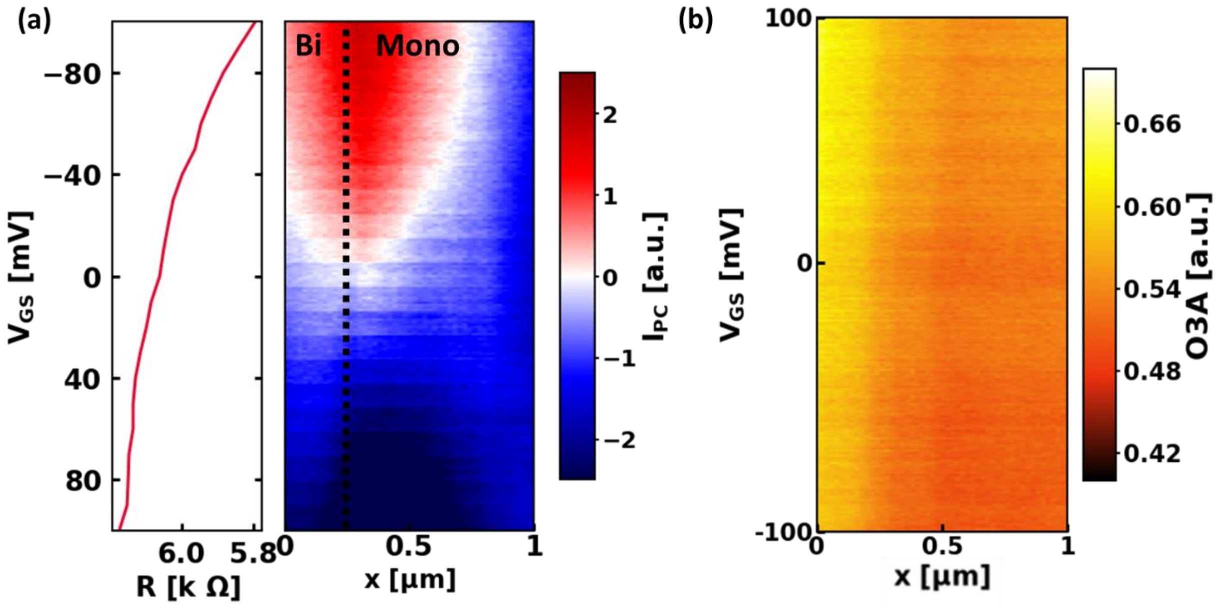

Figure S6: (a) *2nd harmonic photocurrent image measured across the gray line shown in Figure 1e in sample 1 (x-axis), with respect to V_{GS}, ranging from -100 mV to 100 mV in steps of 10 mV, with 10 lines measured for each step. A V_{SD} of 1 mV is applied to simultaneously monitor the resistance, which is shown in dependence of the applied V_{GS} in the graph to the left of the photocurrent maps. The y-axis is shared. The change in sign of the photocurrent at V_{GS}=0 V indicates that the local charge neutrality point (CNP) is approximately at 0 V, with a degree of uncertainty tied to the step size of the applied V_{GS} (10 mV).* ***(b)*** *3rd harmonic optical amplitude recorded simultaneously with (a).*

S7: SD line scans with n far from CNP in Sample 1

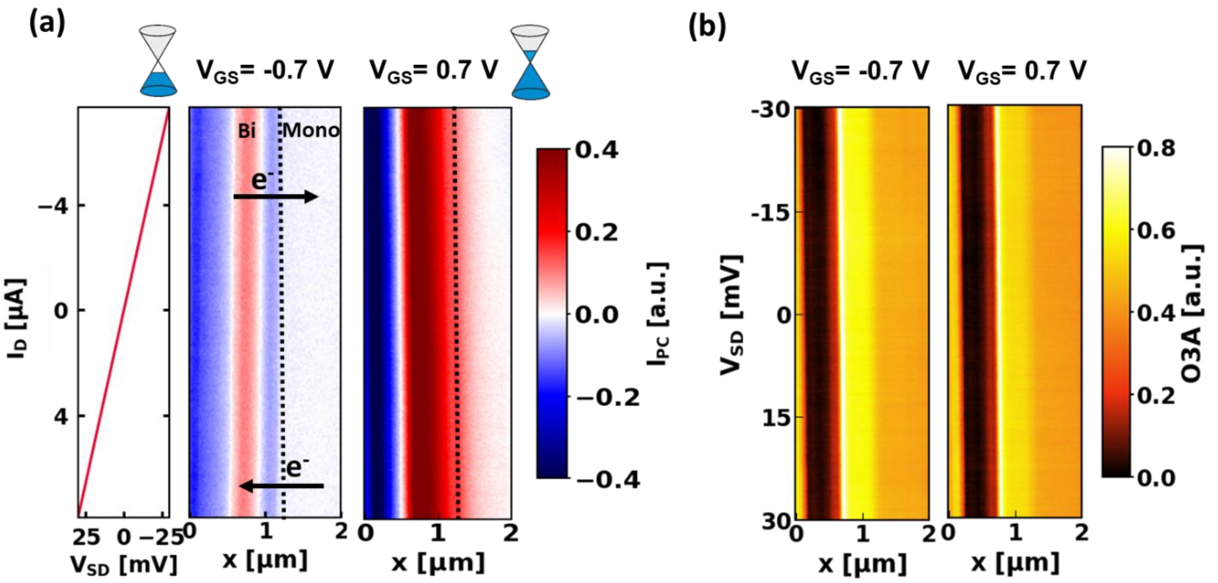

Figure S7: (a) *2nd harmonic photocurrent maps measured along a line across the interface in sample 1 (x-axis), as a function of varying I_D. In the map acquisition the V_{SD} is varied from -30 mV to 30 mV in steps of 2 mV, with 10 lines measured for each step. The two maps are measured for two different V_{GS} values, as indicated by the title on top of each map, V_{GS}=-0.7 V and V_{GS}=0.7 V. These V_{GS} values correspond to charge carrier densities far in the hole and electron doping, as schematically indicated by the Dirac cones at the top of the maps. The graph to the left of the maps shows the SD current with respect to the SD voltage applied, recorded during the photocurrent map taken at V_{GS}=-0.7 V. The y-axis and the color map are shared. The interface is indicated with the dotted line and the arrows indicate the direction of electron flow respectively at the top and bottom halves of the maps (as it depends on the sign of the applied SD).* ***(b)*** *The 3rd harmonic optical amplitude recorded simultaneously to the photocurrent maps. The two optical maps share the same y-axis and color bar.*

In the I_{PC} maps in a) one can see a lack of I_{SD} dependence on the acquired photocurrent signal. It is therefore reasonable to assume that the PTE is the main mechanism generating the I_{PC} in the studied regimes and not the bolometric effect. The bolometric effect is due to the change in resistivity induced by the photo-excitation [14] and is expected to depend on the voltage applied to the sample: its strength is expected to depend on the strength of the field and its polarity on the polarity of the voltage [15]. Although dominant in graphene at high voltage, the bolometric effect is expected to be quite small for the applied electric field at 300 K [16] and it is therefore reasonable to assume that its effect is negligible in the acquired scans.

The oscillating behavior of the photocurrent signal in the map taken at V_{BG}=-0.7 V could be attributed to plasmonic oscillations of the charge carrier density in the BL, initiated by the SNOM tip and reflected by a defect on the left. For highly doped graphene in the Drude regime, it is expected that the near-field interaction between the flake and the SNOM can launch plasmonic waves, which may be detected at the edge of the sample [17–19].These plasmonic oscillations, impacting charge carrier accumulation, could influence the photocurrent response [20], even though they are not clearly evident from the optical amplitude.

S8: SD line scans with n far from CNP in Sample 2

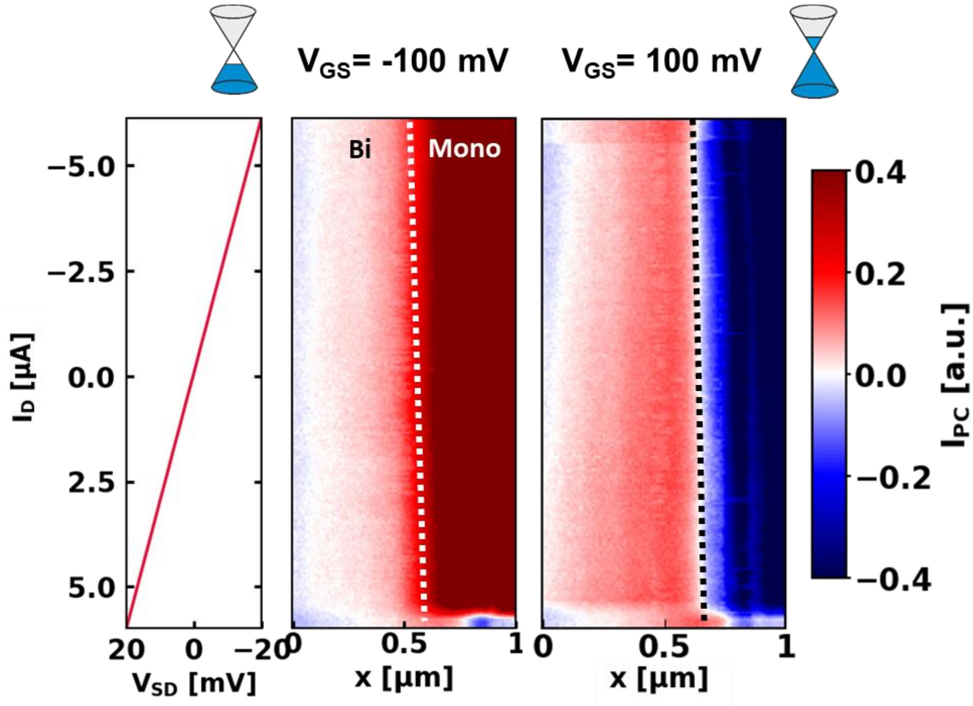

Figure S8: *1st harmonic photocurrent maps measured across the interface in sample 2 (x-axis), as a function of varying I_{SD}. These maps are acquired by varying the V_{SD} from -20 mV to 20 mV in steps of 2 mV, with 10 lines measured for each step. The two maps are recorded for two different V_{GS} values, as indicated by the title on top of each map, V_{GS}=-100 mV and V_{GS}=100 mV. These V_{GS} values correspond to charge carrier densities in the hole and electron region, as schematically indicated by the Dirac cones at the top of the maps. The graph to the left of the maps shows the SD current with respect to the SD voltage applied, recorded during the map taken at V_{BG}= -100 mV. The y-axis and the color bar are shared and the interface is indicated with the dotted line.*

S9: SD line scans around CNP in Sample 2

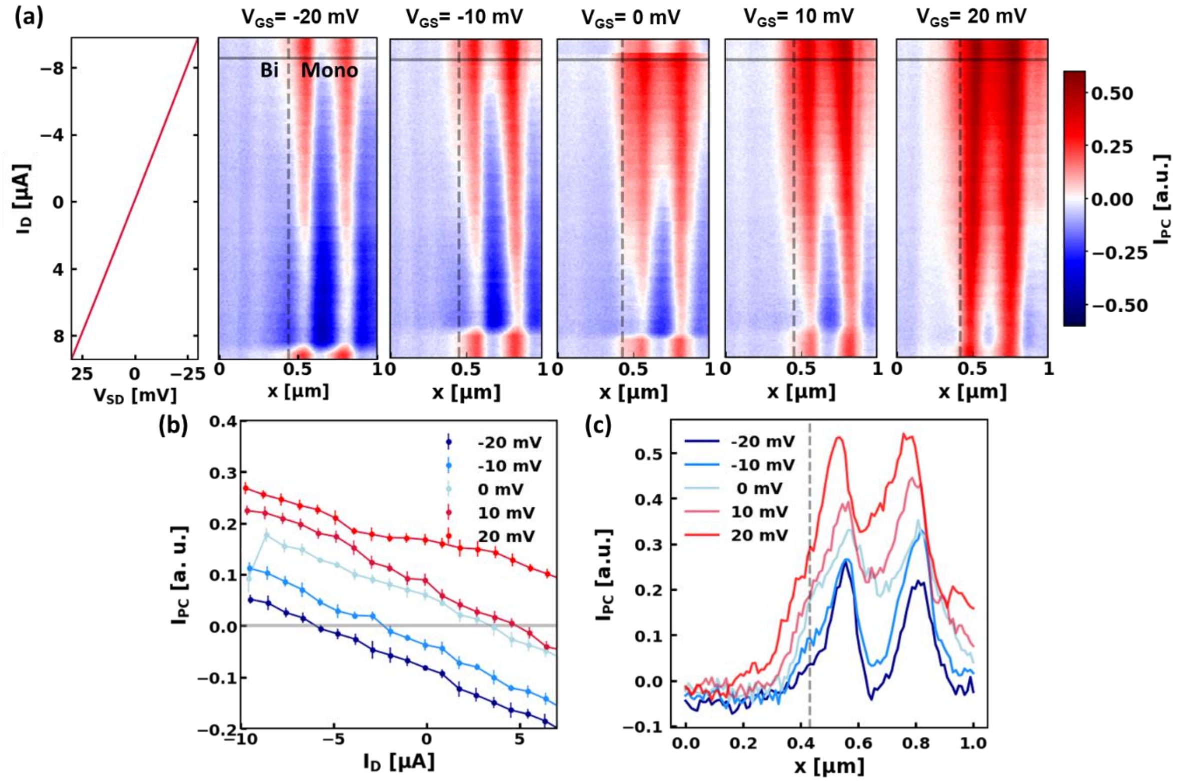

Figure S9: (a) *1st harmonic photocurrent maps measured across the interface in sample 2 (x-axis), as a function of I_D. These maps are acquired varying V_{SD} from -30 mV to 30 mV in steps of 2 mV, with 10 lines measured for each step. The maps are measured for different V_{GS} values, as indicated by the title on top of each map, V_{BG}=-20 mV, V_{GS}=-10 mV, V_{GS}=0 mV, V_{GS}=10 mV and, V_{GS}=20 mV. These V_{GS} are chosen to explore charge carrier densities near the local CNP. The graph to the left of the maps shows the SD current with respect to the SD voltage applied, recorded during the photocurrent map taken at V_{GS}= -20 mV. The graph and the maps share the same y-axis and color bar. The interface is indicated with the dotted line. The photocurrent generated at the interface exhibits a behavior similar to the conic shape predicted in the simulations shown in Figure 4c. It is also important to notice that the two linear features to the left of the interface on the ML side correspond to topographical folds. These inhomogeneities generate a photocurrent that also shows dependence on the SD voltage.* ***(b)*** *Vertical line cuts taken at the interface position of the maps shown in (a), indicated by the dotted lines. For each I_D the average of the 10 I_{PC} values acquired at the dotted line position for that I_D is reported, with their standard deviation shown as an error bar. These cuts compare the I_D dependence of the photocurrent at a fixed x-position across different maps. One can see that the I_D value for which the recorded photocurrent switches sign (i.e. the I_{SD} values crossing the I_{PC}=0 line) depends on the applied V_{GS}, increasing in value for gates further from the local CNP (around 5 mV). This behavior is consistent with the numerical simulation shown in 4c.* ***(c)*** *Horizontal cuts taken along the solid gray lines in the maps shown in (a), comparing the spatial dependence of the photocurrent for different maps at a fixed drain value. The position of the interface is marked by the dashed vertical line. The spatial extent of the photocurrent signal near the interface increases as V_{GS} increases (as it is evident on the BL side). This behavior occurs because the photocurrent sign switch shifts with the applied V_{GS}, so for different V_{GS} values at the same V_{SD}, the cuts intersect different parts of the expected conic shape, with the cone moving downward and its broader base entering the field of view.*

S10: experimental methods

Device Fabrication:

The stacks were fabricated using the dry transfer method described in reference [21]. The graphene and hBN flakes were obtained through mechanical exfoliation, respectively with scotch tape (*Scotch, Magic tape*) and Blue Nitto tape (*Nitto Inc., SWT20+*) onto a Silicon/Silicon dioxide (300 nm) substrate. The graphene flakes were exfoliated either from natural graphite crystals (*NGS trading and consulting*) or a block of highly ordered pyrolytic graphite (*Momentive Performance Materials Inc.*). The hBN flakes were grown by K. Watanabe and T. Taniguchi. The flakes were first initially selected with an optical microscope in bright-field mode (*ZEISS,* model Axio Scope.A1).

The graphene layer thickness is confirmed via Raman spectroscopy. Two different setups were used: a commercially available LabRam HR Evolution (*Horiba)* and a Raman assembly composed of a microscope 100x objective (MPlanFL N 100*, Olympus*) and a Spectrometer iHR550 (*Horiba Scientific*) with a 1800 lines per mm grating. Both are coupled to a 532 nm wavelength laser (torus 532*, Laser Quantum*).

The topography of the flakes of the stack is checked with atomic force microscopy (AFM) before stamping. Three different setups were used: an Asylum Jupiter AFM by *Oxford Instruments*, Dimension Icon (*Bruker)*, all operated with Tap300Al-G (*NanonAndMore*) tips in tapping mode.

An electron beam lithography microscope (*Raith*) operated with an accelerating voltage of 10 kV was used to pattern the electrical contacts. For small contacts, a dose of 110 μC cm^{-2} with a 7.5 μm aperture was utilized, while for bigger contacts and pads a dose of 170 μC cm^{-2} with a 60 μm aperture was used. The layer of resist for the e-beam procedure was obtained following the procedure described in reference [22]. Finally, a 2 nm adhesion layer chromium (with a rate of around 0.43 Å/s) and 60 nm gold contacts (with a rate of around 0.9 Å/s) are evaporated via either thermal evaporation (evaporation chamber from *BesTec*) at pressures of around 10^{-6} mbar or electron-beam physical vapor deposition chamber (electron-beam PVD) at pressures of less than 5x10^{-7} mbar. For one of the two samples studied 1D etch contacts were created by etching through the top hBN flake. For this purpose, an inductive coupled plasma-reactive ion etching (ICP-RIE) device (*Oxford Instruments*, model ICP-RIE Plasmalab System 100) was used. To etch mainly hBN, a mixture of SF6 and Ar was utilized (with a flow rate of 10/5 sccm, an ICP power of 70 W and an RF power of 50 W for an etch rate of 9 nm min^{-1}), while for graphene O2 was utilized (with a flow rate of 10 sccm, an ICP power of 40 W, an RF power of 150 W for an etch rate of 7 nm min^{-1}).

The samples are then glued on a home build chip carrier with a silver conductive paste. The chip carrier is formed by an insulating plastic and contains 18 gold conductive pins, in order to allow electrical

measurements at the SNOM setup. The gold pads are then bonded with a wedge bonder (MEI 1204W*, Marpet Enterprises or* K&S 4500 Series*, Kulicke & Soffa Ltd*) to the gold pins of the chip carrier.

Photocurrent measurements:

The optical near-field scattering microscope and photocurrent images are taken with a commercial s-SNOM (from *Neaspec Company*) coupled to a tunable CO_2 laser (from Access laser, model L4G) with wavelengths of 9.2 µm –10.78 µm. The SNOM is based on an atomic force microscopy (AFM) operated in tapping mode with a tapping amplitude of Δz=90 nm. All the images were taken with two different tips: Arrow-NCPt and Arrow-NCx76-50 (*Nanoworld*), both characterized by a tapping frequency Ω of ~270 kHz. The power of the laser during the measurement was set to around 10 mW.

In order to perform electrical measurements, the s-SNOM is connected to two source measurement units (Keithley 2450, *Tektronix*) and to a current amplifier (DHPCA-100, *FEMTO*) which converts the signal into a voltage and amplifies the AC part of the signal. The output of the amplifier is then transmitted to the DAQ card of the SNOM and the signal is demodulated with the Ω frequency of the tip with an internal lock-in amplifier. For each pixel of a scan, together with the optical signal, two additional values will be recorded, an amplitude A_{PC} and a phase P_{PC} in rad, for all the different harmonics of the tip oscillation frequency Ω. These values are respectively connected to the strength and the direction of the local photocurrent in the system.

The demodulation of the signal to higher harmonics of the tapping frequency enables the isolation of the photocurrent signal derived from the near-field interaction between the tip and the sample from the background signal, dependent on unfocused light, ensuring the nm resolution of the obtained signal. As in typical SNOM images, the optical interaction between the tip and the sample decays exponentially with tip-sample distance, with a steeper decay for higher harmonics and thus the greater the suppression of the background signal. However, higher harmonics also lead to lead to a lower intensity of the acquired signal. In the reported measurements we could not get a signal higher than the third harmonic demodulation order. The second harmonic is primarily analyzed for the sample shown in the main manuscript, while the first-order harmonic is used for the sample presented in the SI, with the general conclusions being the same. In the second sample, the higher harmonic could not be used as its signal was too small.

References

1. Rikhter, A., Basov, D. N. & Fogler, M. M. Modeling of plasmonic and polaritonic effects in photocurrent nanoscopy. *J. Appl. Phys.* **135,** 103101 (2024).

2. Hwang, E. H., Rossi, E. & Das Sarma, S. Theory of thermopower in two-dimensional graphene. *Phys. Rev. B* **80,** 235415 (2009).

3. Castro Neto, A. H., Guinea, F., Peres, N. M. R., Novoselov, K. S. & Geim, A. K. The electronic properties of graphene. *Rev. Mod. Phys.* **81,** 109–162 (2009).

4. McCann, E. & Koshino, M. The electronic properties of bilayer graphene. *Rep. Prog. Phys.* **76,** 56503 (2013).

5. Li, J. *et al.* Effective mass in bilayer graphene at low carrier densities: The role of potential disorder and electron-electron interaction. *Phys. Rev. B* **94,** 161406(R) (2016).

6. Hwang, E. H., Adam, S. & Sarma, S. D. Carrier transport in two-dimensional graphene layers. *Phys. Rev. Lett.* **98,** 186806 (2007).

7. Hwang, E. H. & Das Sarma, S. Screening, Kohn anomaly, Friedel oscillation, and RKKY interaction in bilayer graphene. *Phys. Rev. Lett.* **101,** 156802 (2008).

8. Nam, S.-G., Ki, D.-K. & Lee, H.-J. Thermoelectric transport of massive Dirac fermions in bilayer graphene. *Phys. Rev. B* **82,** 245416 (2010).

9. Zuev, Y. M., Chang, W. & Kim, P. Thermoelectric and magnetothermoelectric transport measurements of graphene. *Phys. Rev. Lett.* **102,** 96807 (2009).

10. Woessner, A. *et al.* Near-field photocurrent nanoscopy on bare and encapsulated graphene. *Nat Commun.* **7,** 10783 (2016).

11. Bae, M.-H., Ong, Z.-Y., Estrada, D. & Pop, E. Imaging, simulation, and electrostatic control of power dissipation in graphene devices. *Nano Lett.* **10,** 4787–4793 (2010).

12. Tielrooij, K.-J. *et al.* Out-of-plane heat transfer in van der Waals stacks through electron-hyperbolic phonon coupling. *Nat. Nanotech.* **13,** 41–46 (2018).

13. Weitz, R. T., Allen, M. T., Feldman, B. E., Martin, J. & Yacoby, A. Broken-symmetry states in doubly gated suspended bilayer graphene. *Science* **330,** 812–816 (2010).

14. Koppens, F. H. L. *et al.* Photodetectors based on graphene, other two-dimensional materials and hybrid systems. *Nat. Nanotech.* **9,** 780–793 (2014).

15. Freitag, M., Low, T., Xia, F. & Avouris, P. Photoconductivity of biased graphene. *Nat. Photon.* **7,** 53–59 (2013).

16. Jago, R., Malic, E. & Wendler, F. Microscopic origin of the bolometric effect in graphene. *Phys. Rev. B* **99,** 35419 (2019).

17. Fei, Z. *et al.* Gate-tuning of graphene plasmons revealed by infrared nano-imaging. *Nature* **487,** 82–85 (2012).

18. Chen, J. *et al.* Optical nano-imaging of gate-tunable graphene plasmons. *Nature* **487,** 77–81 (2012).

19. Xu, Q. *et al.* Effects of edge on graphene plasmons as revealed by infrared nanoimaging. *Light Sci. Appl.* **6,** e16204 (2017).

20. Lundeberg, M. B. *et al.* Thermoelectric detection and imaging of propagating graphene plasmons. *Nat. Mater.* **16,** 204–207 (2017).

21. Winterer, F. *et al.* Spontaneous Gully-Polarized Quantum Hall States in ABA Trilayer Graphene. *Nano Lett.* **22,** 3317–3322 (2022).

22. Lenz, J. *et al.* Charge transport in single polymer fiber transistors in the sub-100 nm regime: temperature dependence and Coulomb blockade. *J. Phys. Mater.* **6,** 15001 (2023).
